# The complete chloroplast genome sequence of *Populus* × *deltoides* L. ‘Jinheiyang’

**DOI:** 10.1080/23802359.2025.2566069

**Published:** 2025-09-29

**Authors:** Yu-Rong Ren, Yong-Hao Nan, Pi-Quan Li, Jian-Zhong Yao, Xiao-Man Yang, Zhi Su

**Affiliations:** ^a^ College of Life Sciences, Hebei University, Baoding, China; ^b^ Shanxi Experiment Bureau for High-Yield poplar Forests, Datong, China; ^c^ Experimental Center of Desert Forestry, Chinese Academy of Forestry, Dengkou, Inner Mongolia, China

**Keywords:** Chloroplast genome, *Populus*, phylogeny analysis

## Abstract

Northwest China's stress-resistant, high-yield poplar *Populus* × *deltoides* L. ‘Jinheiyang’ (JHP) has an unreported chloroplast genome.  This study sequenced it: 157,084 bp circular genome with quadripartite structure, 123 genes (83 protein-coding, 32 tRNA, 8 rRNA), 38.07% GC content. The complete chloroplast genome of JHP may provide basic data for future research on poplar classification and evolution.

## Introduction

Populus L. (Salicaceae) is a globally distributed genus with ∼100 natural species and cultivars across mid-latitudes (Su et al. 2010), Valued for rapid growth and ecological/economic roles in timber production and environmental restoration (Heilman et al. 1999). Chloroplast genomes, characterized by conserved structures and maternal inheritance, serve as robust phylogenetic markers (Olmstead et al. 1994). Although advances in chloroplast and mitochondrial genomics have shed light on plant evolution and nucleocytoplasmic interactions (Daniell et al. 2016; Qu et al. 2023), the phylogeny of poplars remains unresolved due to hybridization and phenotypic complexity (Wang et al. 2023). Populus × deltoides L. ‘Jinheiyang’ (hereinafter referred to as JHP) 2017 is a hybrid cultivar derived from a cross between Populus deltoides cv. Nankang (female parent) and Populus deltoides (male parent) (Zhou 2017). This variety exhibits superior growth characteristics, stress tolerance, and wood quality compared to its parental lines. It is worth mentioning that this variety has strong resistance to Anoplophora glabripennis (the main poplar trunk borer pest in Northwest China. It is an efficient, high-yield, excellent variety suitable for application and promotion in Northwest China. These traits make it critical for ecological restoration in northern China. This paper reports the first complete chloroplast genome assembly map of JHP, aiming to provide basic data for elucidating its genetic structure and adaptive mechanisms, and resolve Populus phylogeny using chloroplast markers (Tao et al. 2016).

## Materials and methods

**Figure 1.**
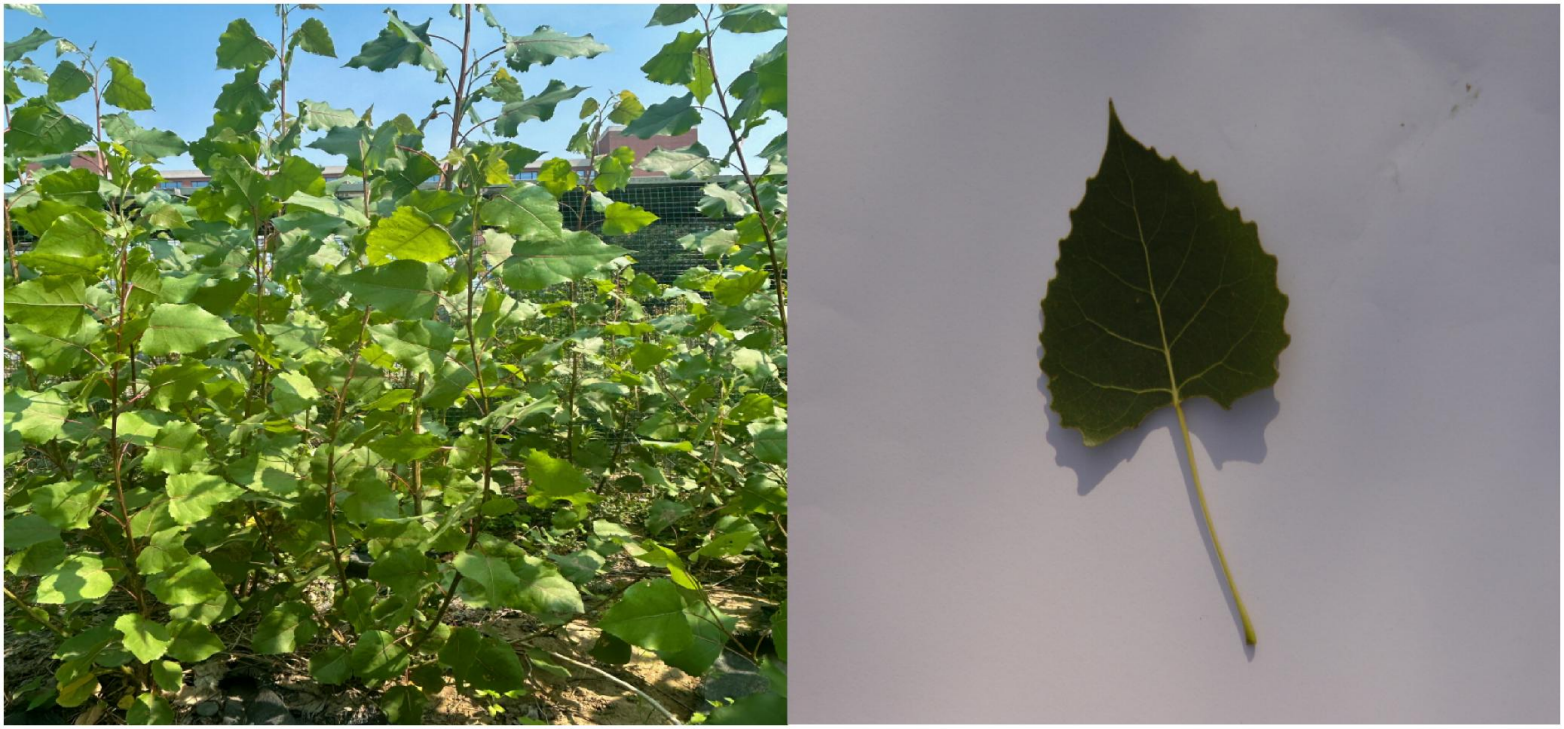
The picture shows the leaves of *Populus × deltoides* L. ‘Jinheiyang’ provided by the Pi-Quan Li and Jian-Zhong Yao. The crown is ovate, the bark is gray when young, gray-brown when old, and has grooves and cracks. The young tree branches and sprouts have obvious ridges and are hairless. The lenticels are nearly round. The buds are short, brown, and sticky. The leaves are triangular.

The Shanxi Experiment Bureau for High-Yield Poplar Forests (112°17′E, 40°19′N) provided fresh cuttings of fresh cuttings, which were potted in the Plant-Insect Interaction Ecology Laboratory of Hebei University (Figure 1). The samples were stored in the Plant-Insect Interaction Ecology Laboratory of the School of Life Sciences of Hebei University (sample voucher number: HBSK402-JHP, contact person: Jian-Rong Wei, weijr@hbu.edu.cn). Genomic DNA was extracted from fresh leaves using a commercial kit (CW2298S, China). Qualified samples were sequenced on an Illumina NovaSeq 6000 using 150 bp paired-end sequencing by Shanghai Applied Protein Technology Co., Ltd. Data screening: Fastq (Chen et al. 2018) software was used to remove paired reads containing adapters, ≥10 bp N bases, or >50% low-quality bases (*Q* ≤ 5), and 9.2 Gb of clean data (GC: 38.07%) were obtained. The genome was assembled using SPAdes software (Bankevich et al. 2012) with *Populus deltoides* (NC_040929) as the reference, and chloroplast genome alignment was performed using CPstools (Huang et al. 2024). Multiple sequence alignment was performed using MAFFT online software (https://mafft.cbrc.jp/alignment/server/; Katoh and Standley 2013). Gblocks (Leduc et al. 2018) was used to filter poorly aligned regions, and IQ-TREE (Nguyen et al. 2014) and FigTree were used to construct the maximum likelihood tree of JHP and 19 other *Populus* species.

In IQ-TREE, the optimal model (K3Pu + F + I + G4) was selected by ModelFinder based on BIC, with 1000 replicates for ultrafast bootstrap and SH-aLRT tests, and node support evaluated using a correlation coefficient threshold of 0.99. Tetrasomal connectivity (Li et al. 2023), nucleotide diversity (pi), and relative synonymous codon usage (RSCU) were analyzed using the Genepioneer platform. tRNA secondary structures were predicted using the online software tRNAscan-SE (http://lowelab.ucsc.edu/tRNAscan-SE) and visualized using VARNA (Darty et al. 2009). Comparative analysis was performed using VISTA software (https://genome.lbl.gov/vista/index.shtml; Frazer et al. 2004), and coverage depth maps were plotted using SAMtools (Li et al. 2009). To guarantee the integrity and accuracy of the assembled chloroplast genome of JHP, it was validated against the chloroplast genomes of *Populus deltoides* (NC_040929), *Populus tomentosa* (NC_040866), and *Populus deltoides* (MT780299) using Geneious software (Kearse et al. 2012). the circular genome map was successfully visualized using OGDRAW (https://chlorobox.mpimp-golm.mpg.de/OGDraw.html).

## Results

**Figure 2.**
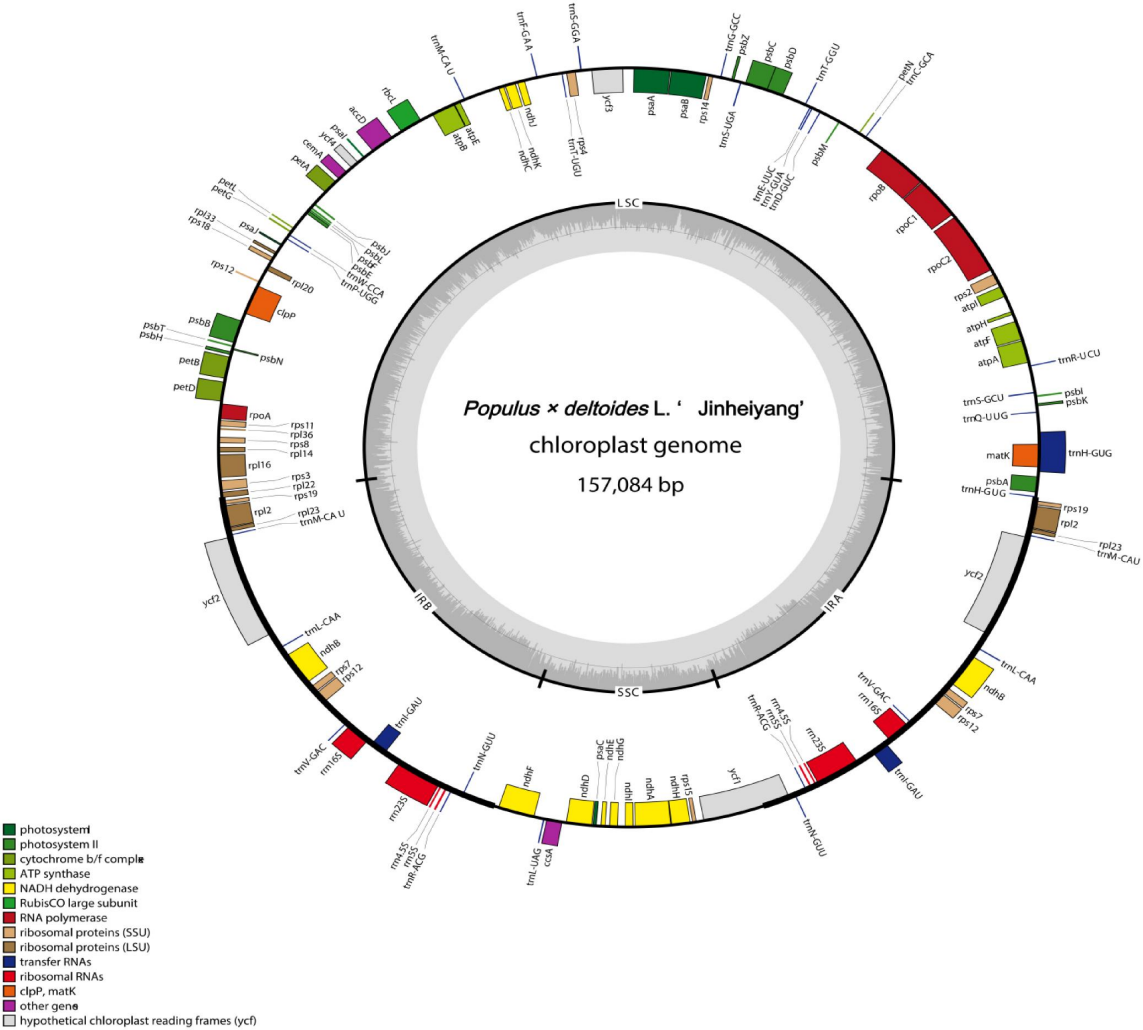
The chloroplast genome map of *Populus* × *deltoides* L. ‘Jinheiyang’. Genes shown outside the circle are transcribed clockwise, and genes inside are transcribed counter-clock-wise. Genes belonging to different functional groups are color-coded. The darker grey in the inner corresponds to the GC content and the lighter grey to the AT content.

**Figure 3.**
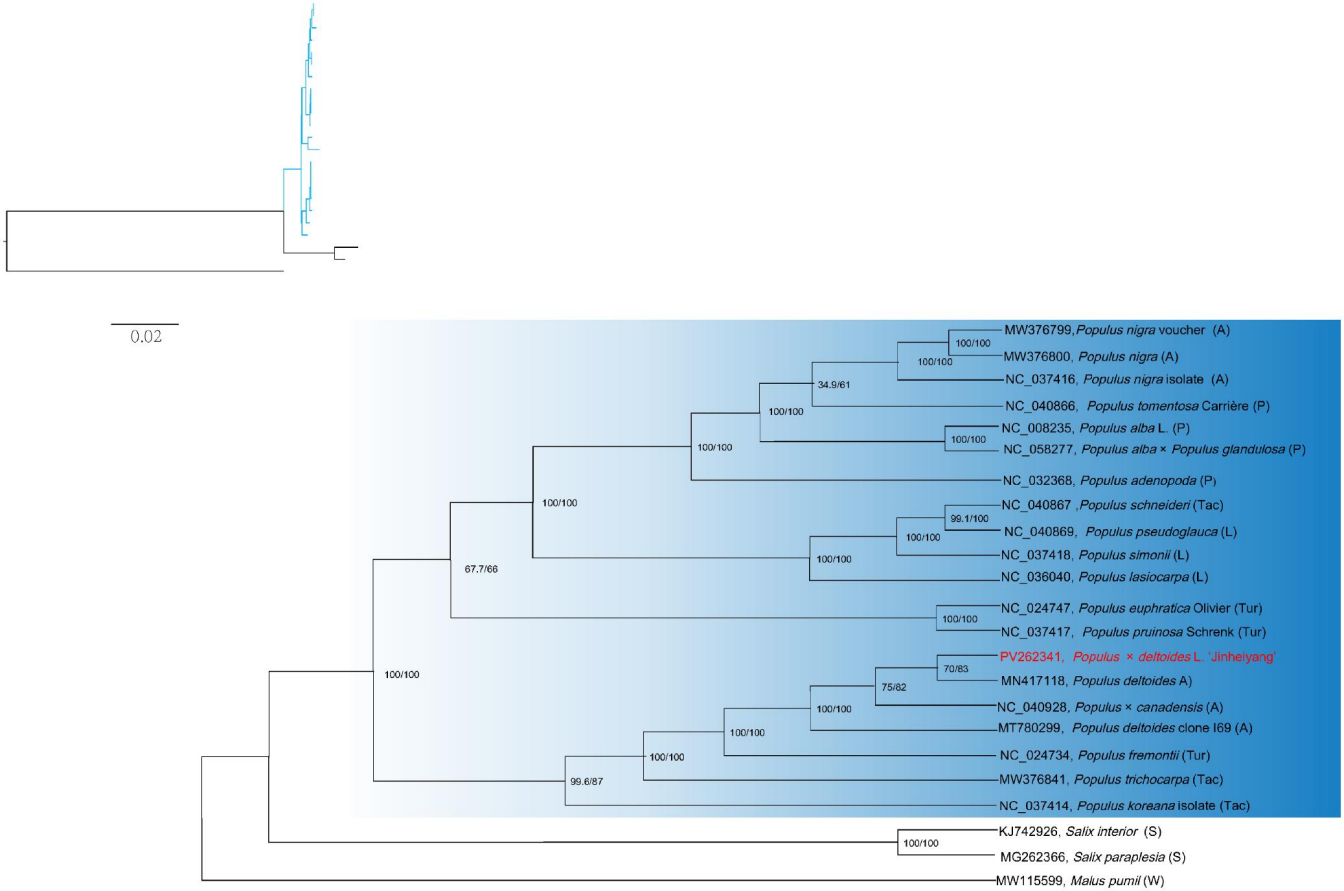
Phylogenetic analysis of *Populus* × *deltoides* L. ‘Jinheiyang’, and other related species based on complete chloroplast genome sequences. The red text logo is JHP. The genome of the species identified with red-bold font is presented in this study. Node numbers represent P, section Leuce; A, section Aigeiros; Tac, section Tacamahaca; L, section Leucoides; Tur, section Turanga; S, Salix; W, Outgroup. In IQ-TREE, the optimal model (K3Pu + F + I + G4) was selected by ModelFinder based on BIC, with 1000 replicates for ultrafast bootstrap and SH-aLRT tests, and node support evaluated using a correlation coefficient threshold of 0.99. The following sequences were used in this study: *Populus alba* × *Populus glan dulosa* NC_058277, *Populus alba* L. NC_008235 (Okumura et al.2006), *Populus tomentosa* Carrière NC_040866 (Zong et al. 2019), *Populus adenopoda* NC_032368 (Zong et al. 2019), *Populus lasiocarpa* NC_036040, *Populus pseudoglauca* NC_040869 (Zong et al.^2019^), *Populus euphratica* Olivier NC_024747 (Zhang and Gao 2016), *Populus pruinosa* Schrenk NC_037417, *Populus deltoides* clone I69 MT780299, *Populus fremontii* NC_024734 (Huang et al.^2014^), *Populus trichocar* MW376841 (Wang et al.^2022^), *Populus nigra* isolate NC_037416*, Populus koreana* NC_037414, *Populus nigra* MW376800 (Wang et al.^2022^), *Populus nigra* voucher MW376799 (Wang et al.^2022^), *Populus deltoides* cultivar (Su et al. 2019), *Populus* × *canadensis* NC_040928 (Zong et al. 2019), *Populus schneideri* NC_040867 (Zong et al.^2019^), *Populus deltoides* MN417118 (Su et al.^2019^), *Populus simonii* NC_037418*, Salix interior* isolate KJ742926 (Huang et al.^2014^), *Salix paraplesia* MG22366, *Malus pumi* MW115599 (Outgroup).

The complete chloroplast genome of JHP has been successfully assembled and annotated. The size of the chloroplast genome is 157,084 bp, which includes two inverted repeat regions (IRs), each spanning 27,649bp. The large single-copy (LSC) region measures 85,096 bp, while the small single-copy (SSC) region is 16,690 bp. The total GC content of the JHP chloroplast genome is 38.07%. The complete chloroplast genome of JHP contains 123 genes, including 83 protein - coding genes, 32 tRNA genes, and 8 rRNA genes (Figure 2). The phylogenetic tree shows that JHP belongs to the Aigeiros species within the Populus, and is highly similar to other tree species within the Aigeiros species, such as *Populus deltoides*, *Populus* × *canadensis*, and *Populus deltoides* clone I69, forming a sister group (Figure 3).

By analyzing the chloroplast genomes of four species (JHP, *Populus deltoides* clone I69, *Populus tomentosa*, *Populus deltoides*) of poplar trees, it was found that there were certain differences in the genomic structure among these four species, for instance, the positions of genes such as *rps19*, *trnN*, *ndhF*, *ycf1* were different (Figure S1). The Pi value distribution is region - specific: the IR region is conserved, while the LSC/SSC region is actively variable (Figure S2). Methionine (Met), Leucine (Leu), and Serine (Ser) are significantly enriched in chloroplast genome - encoded proteins of JHP, reflecting structural conservation of photosynthesis - related proteins and adaptive evolutionary characteristics related to stress resistance (Figure S3). At the genetic level, most genes showed high conservation among the four poplar species, indicating that these genes are relatively stable during evolution and play a crucial role in the survival and growth of poplars (Figure S4). Codon usage of multi - synonymous amino acids (e.g. leucine and arginine) in JHP chloroplast genome shows significant relative synonymous codon usage (RSCU) preference, enhancing adaptability by optimizing translation efficiency of photosynthetic proteins (Figure S5). The sequencing depth values of JHP have a maximum value of 9221×, a minimum value of 27×, and an average value of 6352.79× (Figure S6). Ten genes involving cis-splicing (*atpF*, *rpoC1*, *ycf3*, *clpP*, *petB*, *petD*, *rpl16*, *rpl2*, *ndhB*, *ndhA*) and one gene involving trans-splicing (*rps12*) were identified (Figures S7 and S8).

## Discussion and conclusions

The chloroplast genome of JHP exhibits a typical quadripartite structure (LSC, SSC, IRa/IRb) with a total length of 157,084 bp, encoding 123 genes (83 protein-coding genes, 32 tRNA genes, and 8 rRNA genes) (Supplementary Table 1), which is consistent with the conserved gene content observed in most angiosperm chloroplast genomes (Huang et al. 2014; Zong et al. 2019).

The nucleotide polymorphism (Pi) analysis revealed region-specific conservation: the IR regions are highly conserved, while the LSC and SSC regions show active variability (Supplementary Figure 2). This pattern aligns with the observation that repetitive sequences, which are abundant in noncoding regions of P. deltoides mitochondria (Qu et al. 2023), drive variability in less constrained genomic regions, facilitating adaptive evolution. Additionally, the enrichment of Methionine (Met), Leucine (Leu), and Serine (Ser) in chloroplast-encoded proteins (Supplementary Figure 3) and the significant relative synonymous codon usage (RSCU) preference for multi-synonymous amino acids (e.g. Leucine, Arginine; Supplementary Figure 5) highlight adaptive optimization of translation efficiency for photosynthetic proteins, a phenomenon also linked to functional conservation in mitochondrial genes of angiosperms (Qu et al. 2023).

Overall, this study provides clues for understanding the interspecific diversity of the JHP chloroplast genome, clarifies its systematic status in the Aigeiros faction, offers a new perspective for analyzing the genetic mechanism of chloroplasts in poplar hybrid breeding, and provides basic data for further exploring the molecular evolution and species mechanism of hybrids.

## Supplementary Material

JHY Supplementary Material clean.docx

## Data Availability

The chloroplast genome of *Populus × deltoides* L. ‘Jinheiyang’ in this study is publicly available in NCBI (https://www.ncbi.nlm.nih.gov/) under accession number PV262341. The accession no. of BioProject, Bio-Sample, and SRA are PRJNA1238501, SAMN47474366, and SRR32787063, respectively.
