# Supplementary material for: The complete chloroplast genome sequence of Populus × deltoides L. ‘Jinheiyang’
Source: Mitochondrial DNA B Resour. 2025 Sep 29;10(10):986–90. doi: 10.1080/23802359.2025.2566069 (PMC12486452; doi:10.1080/23802359.2025.2566069)
Supplement: JHY Supplementary Material clean.docx [file TMDN_A_2566069_SM2884.docx]

**Supplementary Table 1.** List of gene in chloroplast genome of *Poplus* × *deltoides* L. ‘Jinheiyang’

| **Gene Functions** | **Group of genes** | **Name of genes** |
| --- | --- | --- |
| Photosynthesis | Subunits of ATP synthase | *atpA*, *atpB*, *atpE*, *atpF**, *atpH*, *atpI* |
|  | Subunits of NADH dehydrogenase | *ndhA**, *ndhB**(×2), *ndhC*, *ndhD*, *ndhE*, *ndhF*, *ndhG*, *ndhH*, *ndhI*, *ndhJ*, *ndhK* |
|  | Subunits of cytochrome | *petA, petB** *,petD***, petG, petL, petN* |
|  | Subunits of photosystem I | *psaA*, *psaB*, *psaC*, *psaI*, *psaJ* |
|  | Subunits of photosystem II | *psbA*, *psbB*, *psbC*, *psbD, psbE*, *psbF*, *psbH*, *psbI, psbJ*, *psbK*, *psbL*, *psbM*, *psbN*, *psbT*, *psbZ* |
|  | Subunit of rubisco | *rbcL* |
| Self-replication | Large subunit of ribosome | *rpl14*, *rpl16**,*rpl2**(×2) *rpl20*, *rpl22*, *rpl23*(×2), *rpl33*, *rpl36* |
|  | Small subunit of ribosome | *rps11*, *rps12***(×2), *rps14*, *rps15*, *rps18*, *rps19(2)*, *rps2*, *rps3*, *rps4*, *rps7*(×2), *rps8* |
|  | DNA dependent RNA polymerase | *rpoA*, *rpoB*, *rpoC1**, *rpoC2* |
|  | Ribosomal RNAs | *rrn16S*(×2), *rrn23S*(×2), *rrn4.5S*(×2), *rrn5S*(×2) |
|  | Transfer RNAs | *trnA-UGC**, *trnC-GCA*, *trnD-GUC*, *trnE-UUC*, t*rnF-GAA*, *trnG-GCC*, *trnH-GUG*, *trnI-GAU**(×2), *trnK-UUU**, *trnL-CAA*(×2), *trnL-UAG, trnM-CAU*(×3), *trnN-GUU*(×2), *trnP-UGG*, *trnQ-UUG*, *trnR-ACG*(×2), *trnR-UCU*, *trnS-GCU*, *trnS-GGA*, *trnS-UGA*, *trnT-GGU*, *trnT-UGU*, *trnV-GAC(2)*, *trnW-CCA*, *trnY-GUA* |
| Other genes | Maturase | *matK* |
|  | Protease | *clpP*** |
|  | Envelop membrane protein | *cemA* |
|  | Subunit of Acetyl-CoA-carboxylase | *accD* |
|  | c-type cytochrome synthesis gene | *ccsA* |
|  | Translational initiation |  |
| Unknown function | Conserved open reading frames | *ycf1*, *ycf2*(×2),*ycf3***, *ycf4* |

Note: *gene with a single intron; **gene with two introns; (×2) duplicated gene.

**Supplementary Table 2.** Phylogenetic tree references

| **Species** | **Genbank** | **Reference** | **DOI** |
| --- | --- | --- | --- |
| *Populus alba* × *Populus glandulosa* | NC_058277. 1 |  |  |
| *Populus alba* L | NC_008235. 1 | Okumura et al., 2006 | 10. 1007/s11248-006-9009-3 |
| *Populus tomentosa* Carrière | NC_040866. 1 | Zong et al., 2019 | 10.3389/fpls.2019.00005 |
| *Populus adenopoda* | NC_032368. 1 | Zong et al., 2019 | 10. 1371/journal.pone.0218455. |
| *Populus lasiocarpa* | NC_036040. 1 |  |  |
| *Populus pseudoglauca* | NC_040869. 1 | Zong et al., 2019 | 10.3389/fpls.2019.00005. |
| *Populus euphratica* Olivier | NC_024747. 1 | Zhang et al.,2016 | 10.3109/19401736. |
| *Populus pruinosa* Schrenk | NC_037417. 1 |  |  |
| *Populus deltoides* clone I69 | MT780299.1 |  |  |
| *Populus fremontii* | NC_024734. 1 | Huang et al.,2014 | 10. 1111/nph.12956. |
| *Populus trichocarpa* | MW376841.1 | Wang et al.,2022 | 10.3389/fpls. |
| *Populus koreana* isolate | NC_037414. 1 |  |  |
| *Salix interior* isolate | KJ742926. 1 | Huang et al.,2014 | 10. 1111/nph.12956. |
| *Salix paraplesia* isolate | MG262366. 1 |  |  |
| *Malus pumila* | MW115599. 1 |  |  |
| *Populus nigra* voucher | MW376799.1 | Wang et al.,2022 | 10.3389/fpls.2022.813177 |
| *Populus nigra* | MW376800.1 | Wang et al.,2022 | 10.3389/fpls.2022.813177 |
| *Populus nigra* isolate | NC_037416.1 |  |  |
| *Populus deltoides* | MN417118.1 | Su et al.,2019 | 10.1080/23802359 |
| *Populus schneideri* | NC_040867 | Zong et al., 2019 | 10.3389/fpls.2019.00005 |
| *Populus simonii isolate* | NC_037418.1 |  |  |
| *Populus × canadensis* | NC_040928 | Zong et al., 2019 | 10.3389/fpls.2019.00005 |

**References**

Huang DI, Hefer CA, Kolosova N, Douglas CJ, Cronk QCB. 2014.Whole plastome sequencing reveals deep plastid divergence and cytonuclear discordance between closely related balsam poplars, *Populus balsamifera* and *P. trichocarpa* (*Salicaceae*).New Phytol. 204(3):693-703.doi: 10. 1111/nph.12956.

Okumura, S., Sawada, M., Park, Y.W., et al. 2006. Transformation of poplar (*Populus alba)* plastids and expression of foreign proteins in tree chloroplasts.Transgenic Res 15, 637–646 . doi:

1. 1007/s11248-006-9009-3.

Su T, Han M, Min J, Cao D, Pan H, Liu Y. 2019. The complete chloroplast genome sequence of *Populus deltoides* 'Siyang-2'. MITOCHONDRIAL DNA B. Dec 13;5(1):283-285.doi: 10.1080/23802359

Wang Y, Huang J, Li E, Xu S, Zhan Z, Zhang X, Yang Z, Guo F, Liu K, Liu D, Shen X, Shang C, Zhang Z. 2022. Phylogenomics and Biogeography of *Populus* Based on Comprehensive

Sampling Reveal Deep-Level Relationships and Multiple Intercontinental .Front Plant Sci. 13:813177.doi: 10.3389/fpls.2022.813177.

Zhang QJ, Gao LZ. 2016. The complete chloroplast genome sequence of desert poplar (*Populus euphratica*).Mitochondrial DNA A DNA Mapp Seq Anal. 721-3. doi:

10.3109/19401736.2014.913159.

Zong D, Gan P, Zhou A, et al. 2019. Plastome Sequences Help to Resolve Deep-Level Relationships of *Populus* in the Family Salicaceae.Front Plant Sci. 10:5.

doi: 10.3389/fpls.2019.00005.

Zong D, Gan P, Zhou A, et al. 2019. Comparative analysis of the complete chloroplast genomes of seven *Populu*s species: Insights into alternative female parents of Populus tomentosa.

PLoS One. 14(6):e0218455.doi: 10. 1371/journal.pone.0218455.

Zong D, Gan P, Zhou A, et al. 2019. Plastome Sequences Help to Resolve Deep-Level Relationships of *Populus* in the Family Salicaceae.Front Plant Sci. 10:5.

doi: 10.3389/fpls.2019.00005.


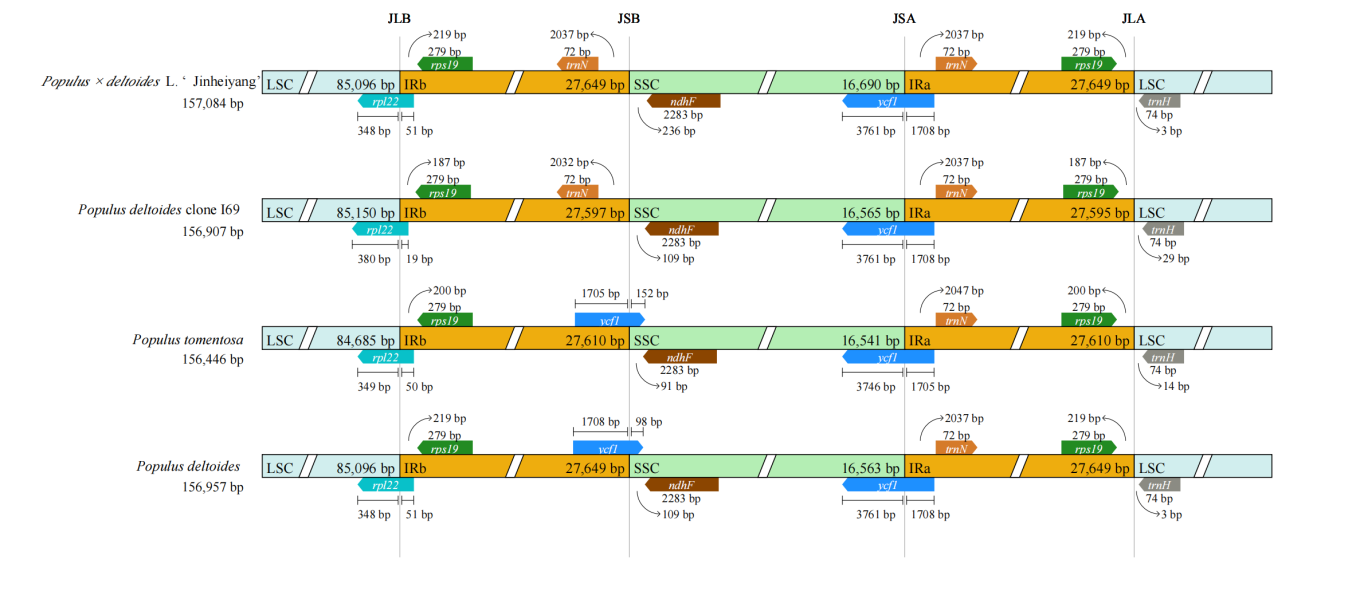


**Supplementary Figure 1.** Chloroplast genome maps of four *Populus* species showing quadripartite structure (LSC ，SSC, IRa/IRb), genome sizes (156,446–157,084 bp), key genes (*rps19*, *trnN*, *ndhF*, *ycf1*), and IR boundary variations.


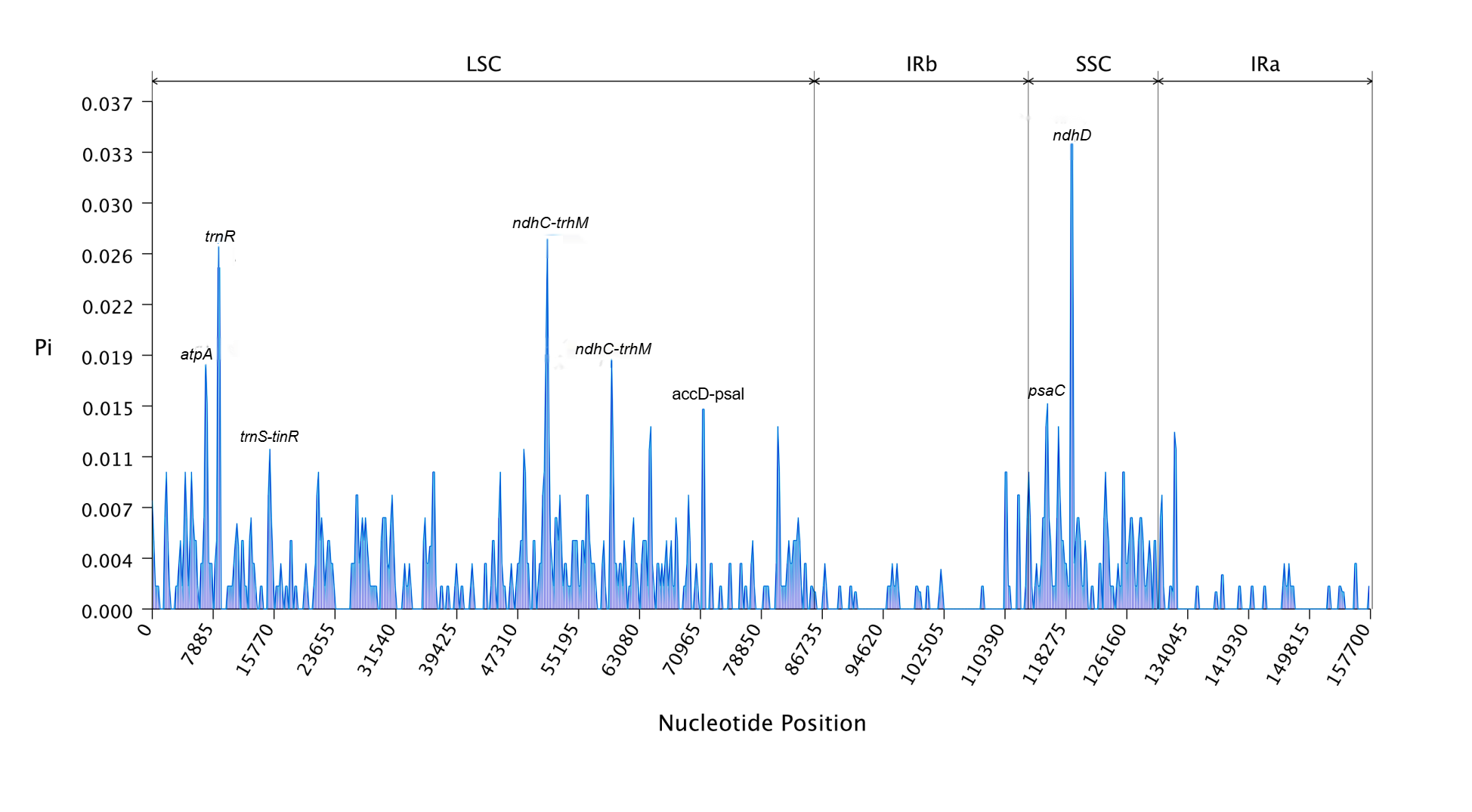


**Supplementary Figure 2.** Nucleotide polymorphism (Pi) distribution across chloroplast genome regions (LSC, IRb, SSC, IRa). The x-axis indicates nucleotide position, while the y-axis shows Pi values with marked gene regions (e.g., *trnR*, *atpA*, *psbD*).


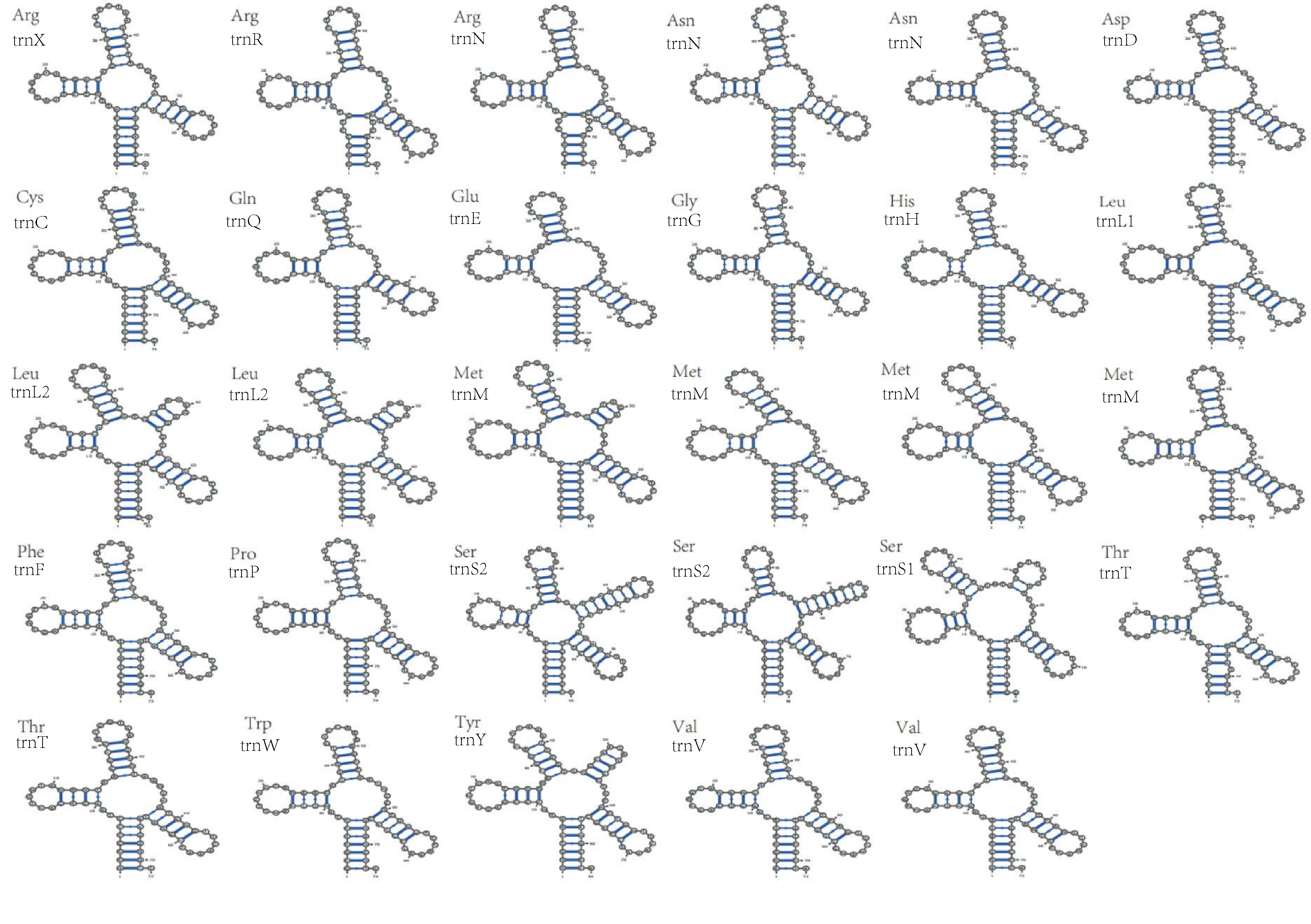


**Supplementary Figure 3.** Secondary structures of tRNAs for various amino acids, labeled with amino acid names and their single-letter codes

(e.g., Arginine (R), Tryptophan (W) ).


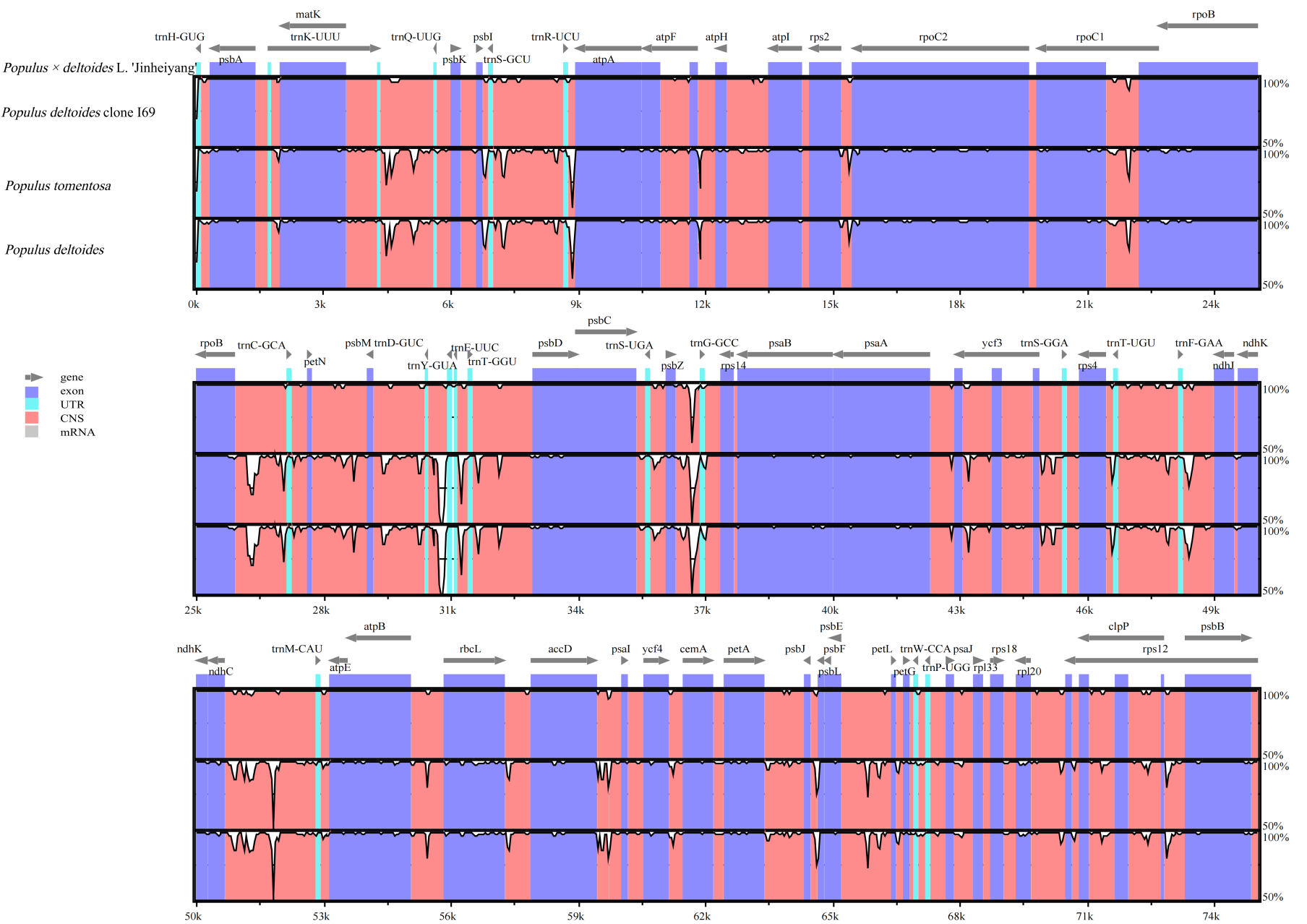


**Supplementary Figure 4.** Genome comparison among *Populus* species ( *Populus* × *deltoides* L. ‘Jinheiyang’, *Populus deltoides* clone I69 , *Populus tomentosa*, *Populus deltoides*), illustrating gene, UTR, CNS, and mRNA regions with sequence similarity percentage.


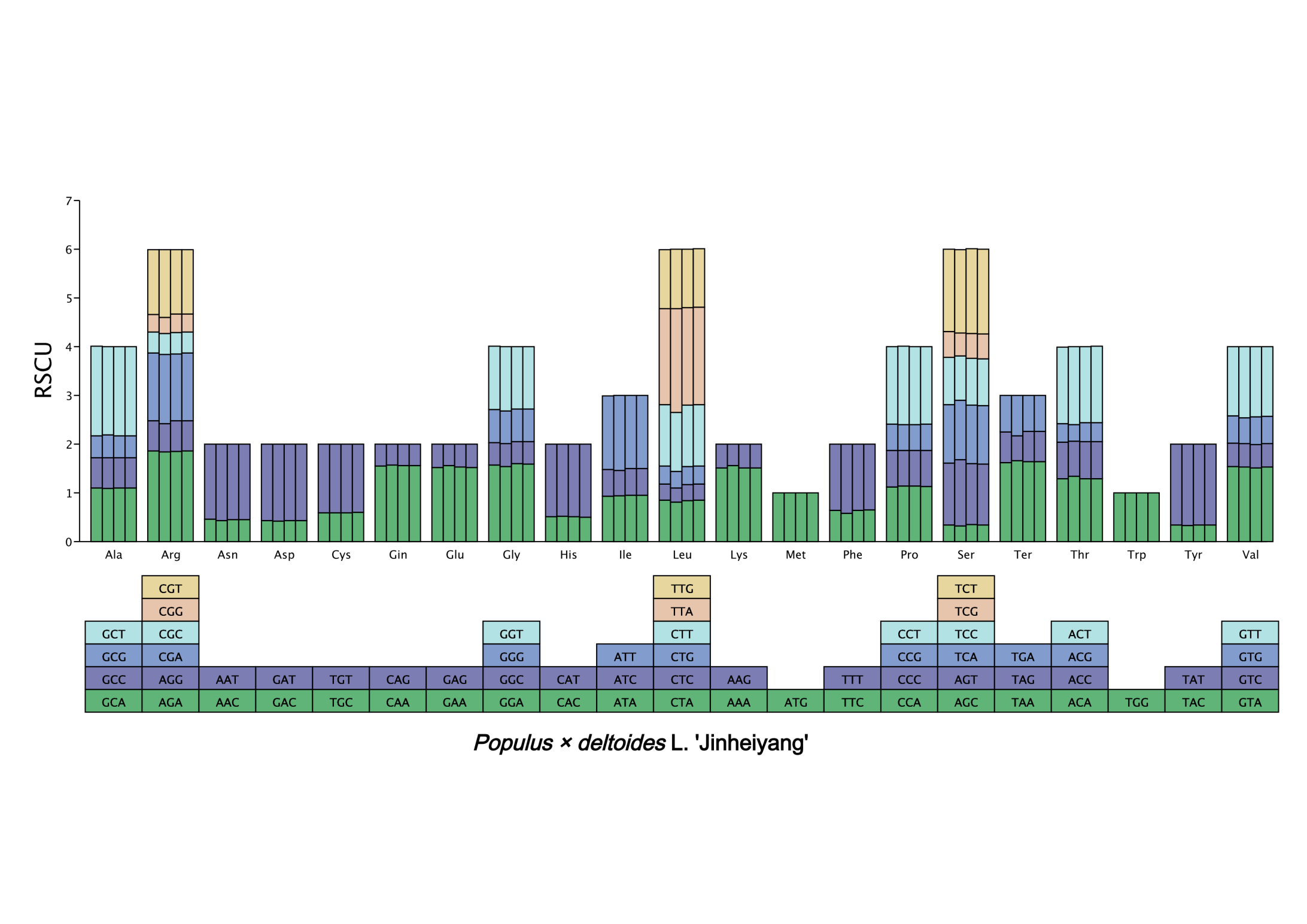


**Supplementary Figure 5**. Relative synonymous codon usage (RSCU) analysis of *Populus* × *deltoides* L. ‘Jinheiyang’. Bars display RSCU values for each amino acid (e.g., Ala, Arg),with color-coded segments representing different codons to illustrate codon usage specificity.


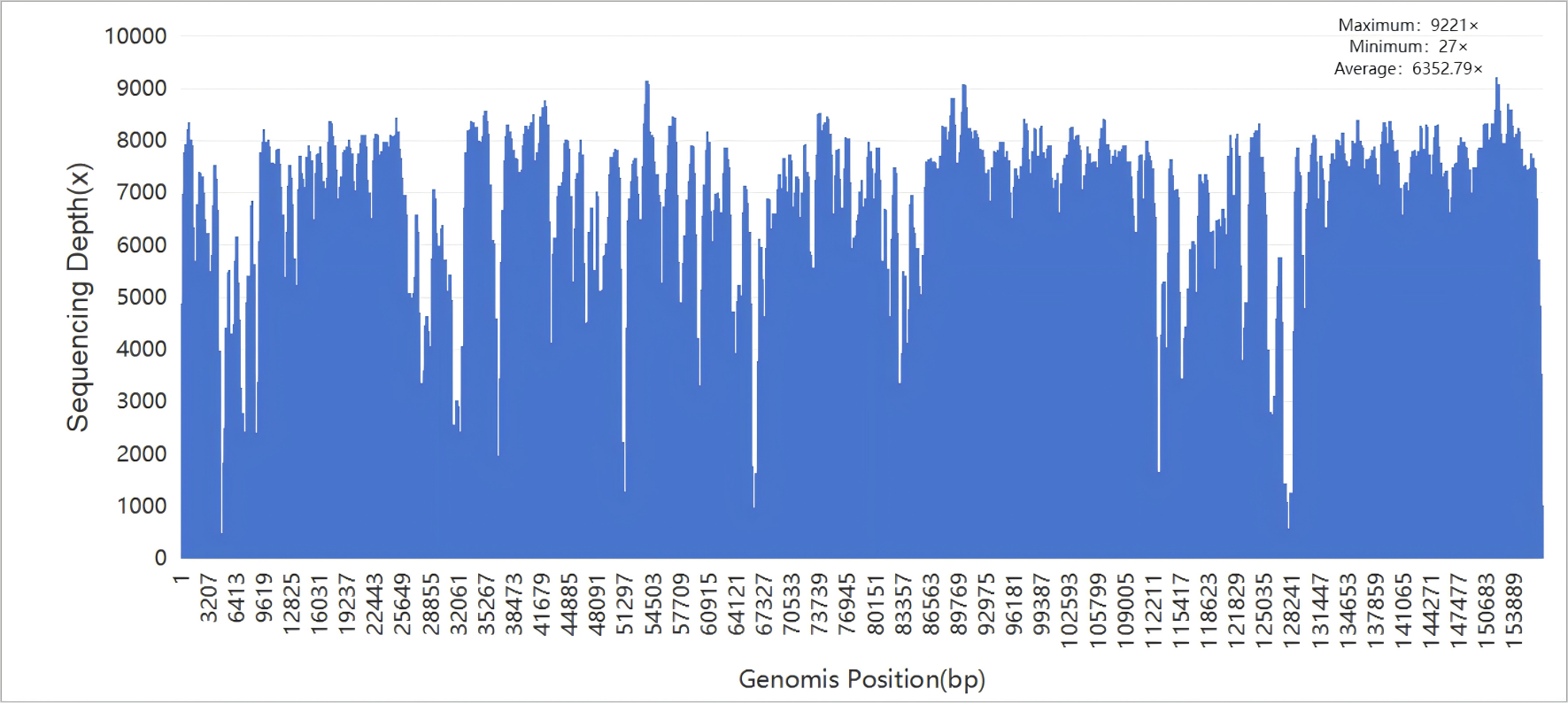


**Supplementary Figure 6.** Sequencing depth distribution at different genomic positions (bp). The maximum sequencing depth is 9221×, the minimum sequencing depth is 27×, and the average sequencing depth is 6352.79×. This figure shows the sequencing depth values across the genome, where the x-axis represents the genomic position and the y-axis represents the sequencing depth level.


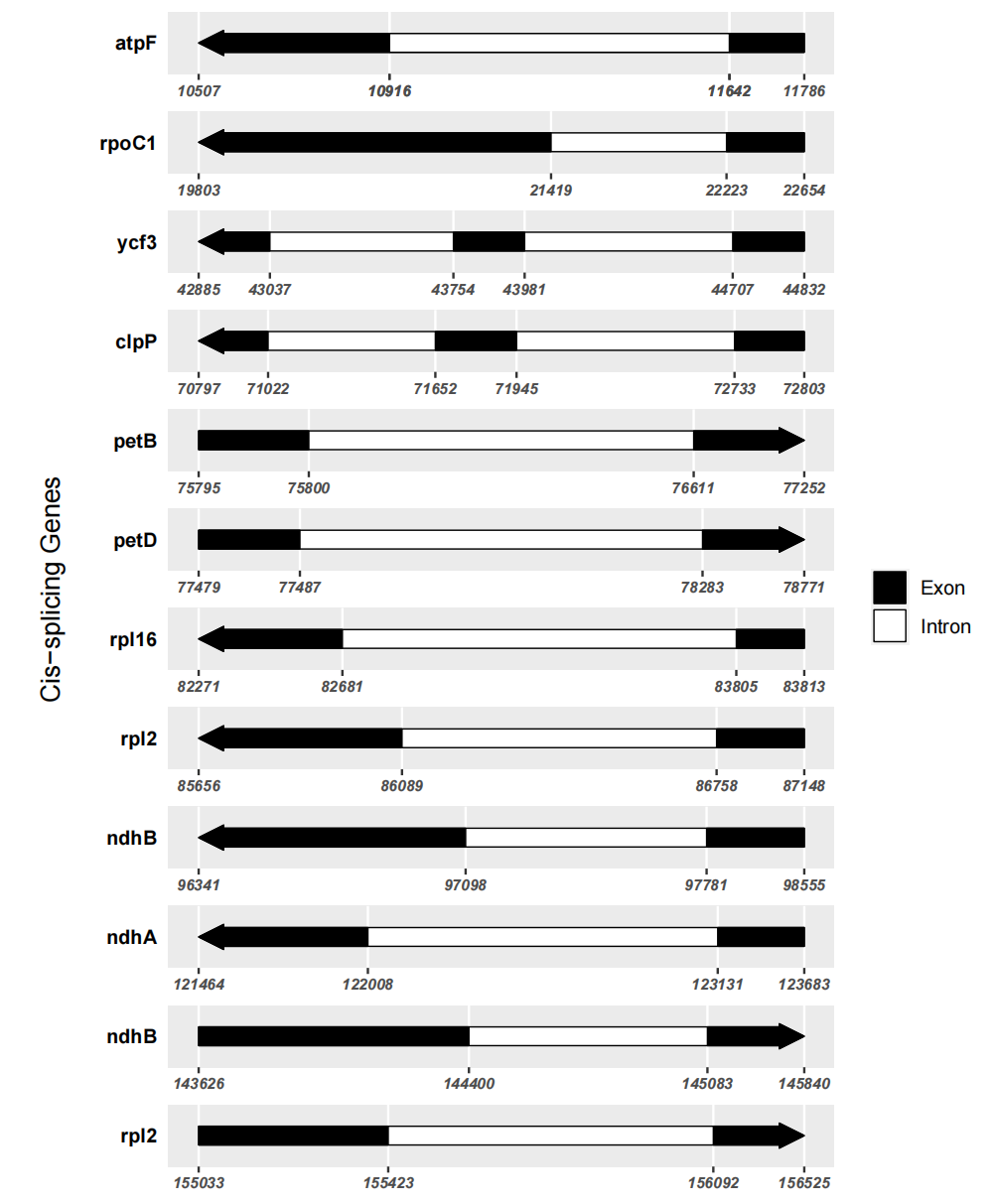
**Supplementary Figure 7.** Depicts a schematic illustration of the cis-splicing genes within the chloroplast

genome. Here, exons are represented in black, while introns are shown in white. An arrow is included to

denote the sense orientation of the gene. Note that the exon and intron lengths are not drawn to scale.


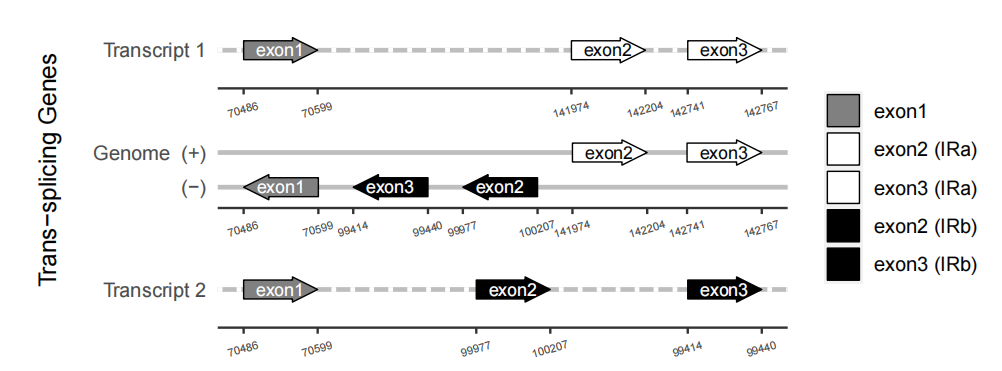


**Supplementary Figure 8.** Trans-splicing gene map of the chloroplast genome (*rps12*) of *Populus* × *deltoides* L. ‘Jinheiyang’.
